# GCN5 inhibits XBP-1S-mediated transcription by antagonizing PCAF action

**DOI:** 10.18632/oncotarget.2773

**Published:** 2014-11-16

**Authors:** Qiao Jing Lew, Kai Ling Chu, Yi Ling Chia, Benjamin Soo, Jia Pei Ho, Chew Har Ng, Hui Si Kwok, Cheng-Ming Chiang, Yao Chang, Sheng-Hao Chao

**Affiliations:** ^1^ Expression Engineering Group, Bioprocessing Technology Institute, Agency for Science, Technology and Research (A*STAR), Singapore; ^2^ Simmons Comprehensive Cancer Center, University of Texas Southwestern Medical Center, Harry Hines Boulevard, Dallas, TX, USA; ^3^ National Institute of Infectious Diseases and Vaccinology, National Health Research Institutes, Tainan, Taiwan; ^4^ Department of Microbiology, National University of Singapore, Singapore

**Keywords:** XBP-1S, UPR, GCN5, PCAF, EBV LMP1

## Abstract

Cellular unfolded protein response (UPR) is induced when endoplasmic reticulum (ER) is under stress. XBP-1S, the active isoform of X-box binding protein 1 (XBP-1), is a key regulator of UPR. Previously, we showed that a histone acetyltransferase (HAT), p300/CBP-associated factor (PCAF), binds to XBP-1S and functions as an activator of XBP-1S. Here, we identify general control nonderepressible 5 (GCN5), a HAT with 73% identity to PCAF, as a novel XBP-1S regulator. Both PCAF and GCN5 bind to the same domain of XBP-1S. Surprisingly, GCN5 potently blocks the XBP-1S-mediated transcription, including cellular UPR genes and latent membrane protein 1 of Epstein-Barr virus. Unlike PCAF, GCN5 acetylates XBP-1S and enhances nuclear retention and protein stability of XBP-1S. However, such GCN5-mediated acetylation of XBP-1S shows no effects on XBP-1S activity. In addition, the HAT activity of GCN5 is not required for repression of XBP-1S target genes. We further demonstrate that GCN5 inhibits XBP-1S-mediated transcription by disrupting the PCAF-XBP-1S interaction and preventing the recruitment of XBP-1S to its target genes. Taken together, our results represent the first work demonstrating that GCN5 and PCAF exhibit different functions and antagonistically regulate the XBP-1S-mediated transcription.

## INTRODUCTION

X-box binding protein 1 (XBP-1) belongs to the cyclic AMP response element binding protein/activating transcription factor (CREB/ATF) family of transcriptional regulators. XBP-1 is an essential factor which controls the terminal differentiation of the antibody-producing plasma cells [1, 2]. Impaired secretion of immunoglobulins is observed in the XBP-1-knockout B cells [1]. XBP-1 also plays a major role in regulating unfolded protein response (UPR), which is triggered when endoplasmic reticulum (ER) is under stress [3]. Two isoforms of XBP-1 are found in cells, XBP-1U and XBP-1S. Both isoforms share a common N-terminus containing a basic-region leucine zipper (bZIP) domain for dimerization and DNA binding. XBP-1U is the dominant isoform under ER stress-free conditions. Activation of UPR induces the endoribonuclease activity of inositol requiring enzyme 1 (IRE1), which removes 26 nucleotides from the open reading frame of XBP-1 mRNA [4]. This unconventional splicing occurs in the cytoplasm and causes a frame shift at amino acid 165 of XBP-1, leading to the generation of XBP-1S by replacing the C-terminus of XBP-1U with a strong transactivation domain [4, 5]. XBP-1U functions as a negative regulator of XBP-1S. XBP-1U interacts with XBP-1S and translocates XBP-1S into the cytoplasm, resulting in proteasome-mediated degradation of XBP-1S [6]. As a transcriptional activator, XBP-1S up-regulates the expression of ER chaperones as well as other genes involved in ER membrane synthesis and protein secretion, suggesting that XBP-1S may serve as an ideal target for cell engineering to enhance production of recombinant secretory proteins [7, 8]. Indeed, we showed that overexpression of XBP-1S increases the secretory capacity of the cell and improves the productivity of recombinant proteins in secretion-limited mammalian cells [9]. Furthermore, we also noticed that nutrient limitations and other environmental stresses of cell culture also induce UPR and generate XBP-1S [10].

Rapid growth of tumor cells coupled with inadequate vascularization leads to shortage of oxygen and nutrients. Both hypoxia and glucose deprivation within solid tumors can induce UPR, resulting in production of XBP-1S [11, 12]. Loss of XBP-1 severely inhibits tumor growth, demonstrating XBP-1 as an essential survival factor for solid tumors [11, 12]. XBP-1S also represents an attractive target for the development of anti-cancer drugs against multiple myeloma (MM), a cancer of plasma cells. Blockade of XBP-1S production using IRE1-inhibiting compounds was found to exhibit significant anti-myeloma activity, suggesting a promising therapeutic option against MM by targeting XBP-1S [13-15].

Infection by more than fifteen different viruses has been reported to induce UPR in the host cells [16-33]. We have demonstrated that XBP-1S regulates gene expression of two oncoviruses, human T-lymphotropic virus type 1 (HTLV-1) and Epstein-Barr virus (EBV) [34, 35]. HTLV-1 is the causative agent of adult T-cell leukemia and lymphoma [36, 37]. The transactivator of HTLV-1, Tax, has been shown to be localized in the organelles associated with protein secretion including ER and Golgi complex [38], raising the possibility that HTLV-1 infection may trigger ER stress and UPR. We found that XBP-1S stimulates basal and Tax-activated transcription of HTLV-1. In addition, infection by HTLV-1 induces UPR and up-regulates the expression of XBP-1, establishing a positive feedback loop between HTLV-1 and the host cells [34]. Nasopharyngeal carcinoma (NPC) is an epithelial malignancy closely associated with EBV [39]. EBV latent membrane protein 1 (LMP1) is a well-documented viral oncoprotein and contributes to development of NPC [40, 41]. We found that expression of LMP1 is induced under UPR and XBP-1S mediates the up-regulation of LMP1 [35]. These studies reveal a role of XBP-1S in regulation of viral transcription and suggest that XBP-1S may also serve as a drug target for development of anti-viral therapeutics.

The localization of a transactivation domain within the C-terminus of XBP-1S helps to explain the transactivating ability of XBP-1S. In our previous study, we identified a histone acetyltransferase (HAT), p300/CBP-associated factor (PCAF), as a specific binding protein and an activator of XBP-1S through the interaction with the transactivation domain of XBP-1S [42]. However, the molecular mechanism of XBP-1S transactivation remains largely unknown and additional factors may be required for regulation of XBP-1S activity. Here we discover another HAT, general control nonderepressible 5 (GCN5), as a novel XBP-1S binding protein. Although sharing 73% identity with PCAF and associating with the transactivation domain of XBP-1S, GCN5 unexpectedly demonstrates opposite effects on XBP-1S and inhibits XBP-1S-mediated transcription. Our results demonstrate a novel function of GCN5 in UPR by modulating the activity of XBP-1S.

## RESULTS

### The C-terminal transactivation domain of XBP-1S interacts with GCN5

**Figure 1:**
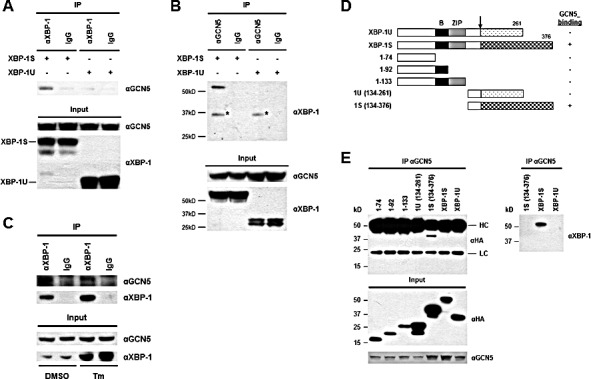
XBP-1S interacts with GCN5 through its specific C-terminal region (A) 293T cells were co-transfected with a GCN5 and an indicated XBP-1 expression plasmid (XBP-1U or XBP-1S). IP was performed by incubating the cell lysates prepared from the transfected cells with anti-XBP-1 (A) or anti-GCN5 (B) antibodies. Normal IgG (IgG) was used as a negative control and non-specific protein bands were marked with asterisks. The immunoprecipitated complexes and the protein inputs were analyzed by Western blotting. (C) Nuclear extracts prepared from cells treated with or without Tm were analyzed IP using an anti-XBP-1 antibody. Tm was dissolved in DMSO and the final concentration of DMSO in the culture was kept at 0.1%. Cells treated with 0.1% DMSO were served as a negative control. The immunoprecipitated complexes and the protein inputs were analyzed by Western blotting. (D) Diagram of XBP-1 truncations. All the constructs were HA-tagged. *B*, basic domain; *ZIP*, leucine zipper domain. (E) 293T cells were co-transfected with a GCN5 and an indicated plasmid to express an individual XBP-1 deletion. IP was performed using the anti-GCN5 antibody followed by Western blotting with anti-HA or anti-XBP-1 antibodies.

We previously demonstrated that PCAF interacts with the transactivation domain of XBP-1S and activates XBP-1S-mediated transcription [42]. Knockdown of PCAF only partially blocks the expression of XBP-1S target genes, suggesting the possible requirement of other factors for XBP-1S-dependent transcription [42]. Another HAT, GCN5, which shares 73% identity with PCAF (in amino acid sequences) [43], is a potential candidate for this transcriptional regulation. We thus examined the interaction between GCN5 and two XBP-1 isoforms, XBP-1U and XBP-1S. Cells were co-transfected with GCN5 and XBP-1 (i.e., XBP-1U or XBP-1S) expression plasmids followed by Co-immunoprecipitation (Co-IP) analysis (Figure 1A). The anti-XBP-1 antibody used in the assays recognizes both XBP-1 isoforms. GCN5 was found in the immunoprecipitated complexes of XBP-1S-expressing cells, but not in XBP-1U-transfected cells (Figure 1A). Reciprocal IP using an anti-GCN5 antibody showed that XBP-1S was detected in the immunoprecipitated complexes, confirming the interaction between GCN5 and XBP-1S (Figure 1B). We further examined the interaction between endogenous XBP-1S and GCN5 proteins with or without UPR induction. A UPR inducer, tunicamycin (Tm), was used to trigger UPR in cells. Since the protein level of XBP-1S in the ER stress-free cells was extremely low, we carried out IP using nuclear extracts prepared from Tm-treated and untreated cells. An increase in the protein level of XBP-1S was detected in the Tm-treated cells (Figure 1C, input). As shown in Figure 1C, we confirmed the association between endogenous XBP-1S and GCN5 proteins. Interestingly, decreased XBP-1S-GCN5 interaction was detected once UPR was induced [Figure 1C, dimethyl sulfoxide (DMSO) vs Tm treatment].

To narrow down the specific region of XBP-1S involved in GCN5 binding, we carried out domain mapping using a series of hemagglutinin (HA) tagged XBP-1 truncations (Figure 1D). Cells were co-transfected with a GCN5 vector and an individual XBP-1 truncation plasmid followed by IP using an anti-GCN5 antibody. Only the XBP-1S-specific C-terminal region, which contained the transcriptional activation domain of XBP-1S, was found to associate with GCN5 (Figure 1E). Neither the XBP-1U-specific C-terminus nor any other domains of XBP-1 interacted with GCN5 (Figure 1E). We noticed that the heavy chains of anti-GCN5 antibodies were also recognized by the secondary antibody used for the immunoblotting. Since the molecular weights of heavy chains and HA-tagged XBP-1S were similar (~50 kD), the blot could not reveal the presence of HA-XBP-1S in the immunoprecipitates. To confirm the interaction between GCN5 and HA-XBP-1S in this set of experiments, we did another Western blot using an anti-XBP-1 antibody recognizing the common domain of XBP-1S and XBP-1U (Figure 1E, the anti-XBP-1 blot). Collectively, the results demonstrate that GCN5 binds to XBP-1S through the transcriptional activation domain of XBP-1S located in its C-terminal region. This is the same XBP-1S domain where PCAF associates with [42].

**Figure 2:**
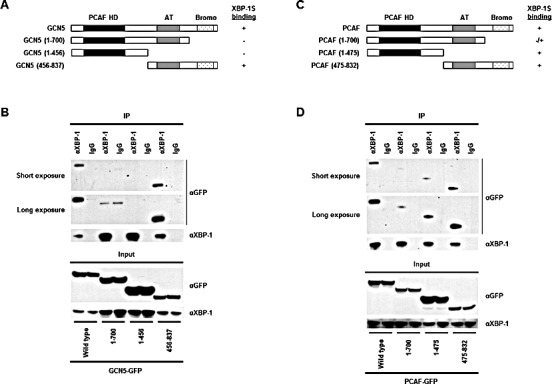
Domain study of GCN5 and PCAF (A) Diagram of GCN5 truncations. All the constructs were GFP-tagged. *PCAF HD*, PCAF homology domain; *AT*, acetyl transferase domain; *Bromo*, bromo domain. (B) 293T cells were co-transfected with a XBP-1S and an indicated plasmid to express an individual GCN5 deletion. IP was performed using the anti-XBP-1 antibody followed by Western blotting with anti-GFP or anti-XBP-1 antibodies. Normal IgG (IgG) was used as a negative control. (C) Diagram of PCAF truncations. All the constructs were GFP-tagged. (D) 293T cells were co-transfected with a XBP-1S and an indicated plasmid to express an individual PCAF deletion. IP was performed using the anti-XBP-1 antibody followed by Western blotting with anti-GFP or anti-XBP-1 antibodies.

We continued to map the domains of GCN5 and PCAF required for XBP-1S interaction. The C-terminal region of GCN5, containing the acetyl transferase and bromo domains, was found to bind to XBP-1S (Fig. 2A and B, 456-837 a.a.). No interaction between GCN5 and XBP-1S was detected when the bromo domain was deleted (Fig. 2A and B, 1-700 a.a.). Compared to GCN5, PCAF exhibited a different binding mechanism with XBP-1S. Both N- and C-terminal domains of PCAF were able to associate with XBP-1S (Fig. 2C and D, 1-475 and 475-832 a.a.). Furthermore, deletion of the bromo domain of PCAF did not completely abolish the PCAF-XBP-1S interaction (Fig. 2C and D, 1-700 a.a.). Such differences in binding between GCN5/PCAF and XBP-1S imply that GCN5 and PCAF may exhibit distinct effects on the biological function of XBP-1S.

### GCN5 inhibits XBP-1S-mediated UPR gene and EBV LMP1 expression

**Figure 3:**
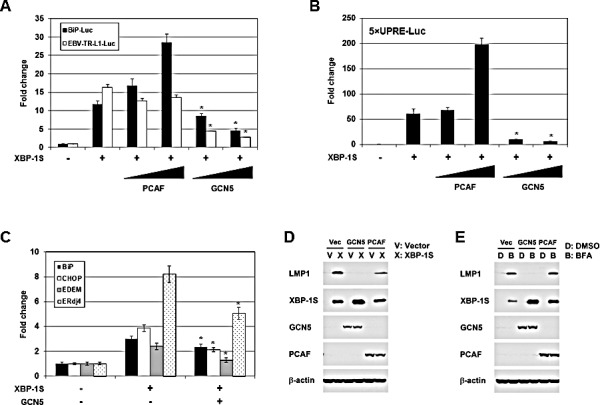
GCN5 negatively regulates XBP-1S-mediated transcription (A) HEK293 cells were transiently co-transfected with a luciferase reporter (BiP-Luc or EBV-TR-L1-Luc) and indicated expression plasmids (i.e. XBP-1S, PCAF, and GCN5). The amounts of PCAF and GCN5 plasmids were titrated at 3-fold increment. Cells transfected with an empty vector were used as a negative control. The total amounts of plasmids transfected were kept constant by adjusting the mock vector. *P<0.05 vs control (i.e. cells transfected with the XBP-1S expression plasmid). (B) HEK293 cells were transiently co-transfected with a 5×UPRE-Luc reporter and indicated expression plasmids (i.e. XBP-1S, PCAF, and GCN5). The amounts of PCAF and GCN5 plasmids were titrated at 3-fold increment. Cells transfected with an empty vector were used as a negative control. *P<0.05 vs control (i.e. cells transfected with the XBP-1S expression plasmid). (C) MCF7 cells were co-transfected with the indicated plasmid (i.e. empty, XBP-1S, or GCN5 expression vectors). The mRNAs of the XBP-1S target genes, including BiP, CHOP, EDEM, and Erdj4 were quantified by qRT-PCR. Cells transfected with an empty vector served as a negative control. *P<0.05 vs control (i.e. cells transfected with a XBP-1S expression plasmid). (D) NPC-TW01/EBV cells were co-transfected with the expression plasmids as indicated (Vec or V: an empty vector). Expression of LMP1, XBP-1S, GCN5, PCAF, and β-actin was analyzed by Western blotting 2 days post-transfection. (E) NPC-TW01/EBV cells were co-transfected with the indicated vectors. One day after transfection, the transfected cells were treated with an ER stress inducer brefeldin A (BFA, 0.1 μg/ml) for one more days, followed by Western blot analysis.

Functional significance of the GCN5-XBP-1S interaction was assessed in XBP-1S-dependent transcription assays. XBP-1S is known to regulate the transcription of cellular gene BiP and EBV oncogene LMP1 [7, 35]. The luciferase reporters, in which the expression of luciferase was driven by BiP and EBV LMP1 promoters (i.e. BiP-Luc and EBV-TR-L1-Luc, respectively), were utilized in the study. In the XBP-1S co-transfected cells, more than 10-fold increases in luciferase expression were observed in BiP and EBV LMP1 promoters (Figure 3A). In agreement with our previous study, PCAF activated the XBP-1S-mediated BiP transcription (Figure 3A) [42]. However, PCAF failed to further stimulate the XBP-1S-mediated activation of EBV LMP1 promoters (Figure 3A). Unexpectedly, GCN5 significantly decreased the luciferase expression driven by either BiP or EBV LMP1 promoters (Figure 3A). XBP-1S regulates the transcription of its target genes by binding to the UPR element (UPRE) located within their promoters [7, 44]. We next determined if transcriptional repression of XBP-1S target genes by GCN5 was mediated through UPRE as well. To address this issue, we utilized a luciferase reporter vector, 5×UPRE-Luc (which contains five UPREs), for the cell-based assay [44, 45]. In XBP-1S-expressing cells, more than 50-fold increases in luciferase expression were observed and co-transfection of PCAF further activated the XBP-1S-dependent transcription (Figure 3B). In contrast, overexpression of GCN5 caused more than 5-fold inhibition of the XBP-1S-mediated Luc expression driven by UPREs (Figure 3B). These results suggest that repression of the XBP-1S-mediated transcription by GCN5 is UPRE-dependent.

Requirement of GCN5 for the expression of endogenous XBP-1S target genes, including BiP, CHOP, EDEM, and ERdj4 [7], was investigated by quantitative reverse transcriptase-polymerase chain reaction (qRT-PCR). Overexpression of XBP-1S resulted in 2.5- to 8-fold increases in the mRNA levels of BiP, CHOP, EDEM, and ERdj4 (Figure 3C). Expression of these XBP-1S target genes was significantly inhibited when GCN5 was co-expressed in the cells (Figure 3C). We continued to examine the impact of GCN5 on the EBV LMP1 oncogene. As reported previously, overexpression of XBP-1S in EBV-infected NPC-TW01 cells induced the expression of LMP1 [35]. Co-expression of GCN5 was found to completely inhibit the XBP-1S-activated-LMP1 expression, while PCAF partially blocked the synthesis of LMP1 (Figure 3D). Similar results were also observed in the Luc reporter assays performed earlier (Figure 3A). An alternative approach was utilized to activate the XBP-1S-mediated expression by treating the EBV-infected NPC-TW01 cells with brefeldin A (BFA), an ER stress inducer. Induction of XBP-1S as well as LMP1 was observed in the BFA-treated cells (Figure 3E). Once again, co-expression of GCN5 completely blocked the LMP1 synthesis, while no effect was detected when PCAF was overexpressed in the cells (Figure 3E).

**Figure 4:**
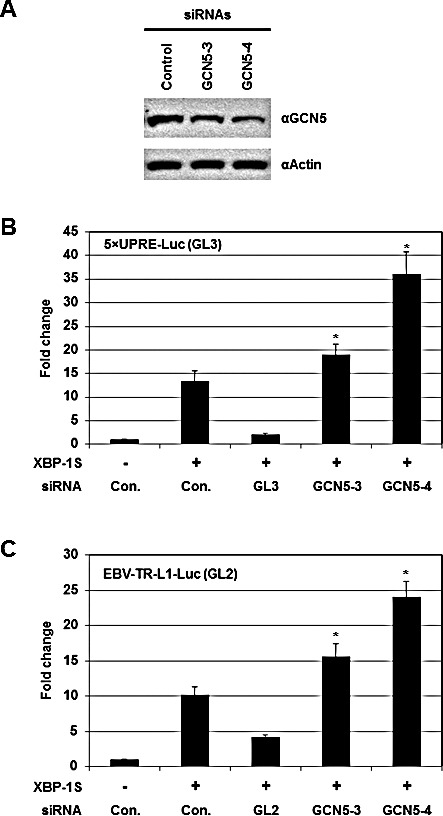
Knockdown of GCN5 stimulates XBP-1S-mediated transcription (A) Cell lysates of the GCN5 siRNA-transfected 293T cells were analyzed by Western blotting to determine the effectiveness of the siRNAs. Cells transfected with a non-specific siRNA were used as a negative control (i.e. Control). For the luciferase-based assays, 293T cells were transiently co-transfected with a luciferase reporter [(B) 5×UPRE-Luc (GL3) or (C) EBV-TR-L1-Luc (GL2)], a XBP-1S expression plasmid, and an indicated siRNA. The siRNAs used for the experiments included non-specific (i.e. Con.), luciferase (i.e. GL3 or GL2), and two GCN5 (i.e. GCN5-3 and GCN5-4) siRNAs. *P<0.05 vs control (i.e. cells co-transfected with a control non-specific siRNA and a XBP-1S expression plasmid).

Knockdown experiments were carried out using siRNAs specifically targeting GCN5. We first confirmed the effectiveness of two GCN5 siRNAs, GCN5-3 and GCN5-4, by Western blotting (Figure 4A). Cells were then co-transfected with a luciferase reporter (i.e. 5×UPRE-Luc or EBV-TR-L1-Luc), a XBP-1S expression plasmid, and an indicated siRNA (Figures 4B and C). Compared to the transfection with an empty vector, 12- and 10-fold enhancement in the activation of 5×UPRE and EBV-TR-L1 promoters was observed in the XBP-1S expressing cells (Figures 4B and C, the first two transfections). The GL3 and GL2 siRNAs, which specifically targeted the luciferase used in the 5×UPRE-Luc and EBV-TR-L1-Luc reporters, respectively, was used as positive controls and caused more than 50% decreases in luciferase expression (Figures 4B and C, the second and third transfections). The two GCN5 siRNAs further significantly activated the luciferase expression driven by both promoters (1.5- to 3-fold; Figures 4B and C). In agreement with the overexpression assays, these results demonstrated GCN5 as a repressor of XBP-1S-mediated transcription. We continued to examine the effects of knocking down endogenous GCN5 on the expression of endogenous XBP-1S target genes using the more effective GCN5 siRNA, GCN5-4. Up-regulation of two XBP-1S target genes, BiP and EDEM, was observed when endogenous GCN5 was knocked down (Figure S1).

### GCN5 is involved in the regulation of XBP-1S target genes during UPR

**Figure 5:**
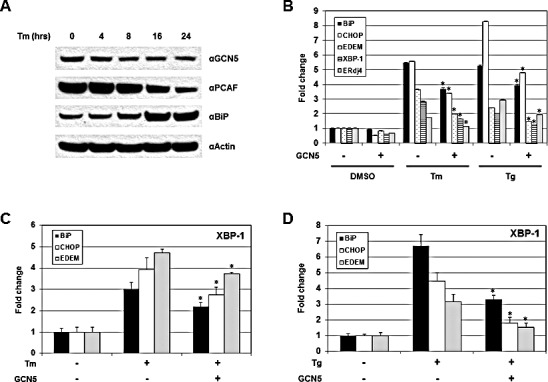
GCN5 overexpression hampers UPR *in vivo* (A) 293T cells were treated with 10 μg/ml Tm for the indicated time. Expression of GCN5, PCAF, BiP, and actin were analyzed by Western blotting. (B) Cells were transfected with a mock or a GCN5 expression vectors, followed by Tm (10 μg/ml, 16 hrs) or Tg (300 nM, 4 hrs) treatment. Both Tm and Tg were dissolved in DMSO and the final concentration of DMSO in the culture was kept at 0.1%. Cells treated with 0.1% DMSO were served as a negative control. Expression of endogenous BiP, CHOP, EDEM, XBP-1, and ERdj4 genes was determined by qRT-PCR. Cells were transfected with an empty or a GCN5 plasmid followed by Tm (C) or Tg (D) treatment. ChIP-quantitative-PCR assays were carried out to quantify the abundance of XBP-1S located on the promoters of BiP, CHOP, and EDEM genes. *P<0.05 vs controls (i.e. cells treated with Tm or Tg, respectively).

The role of GCN5 under UPR was investigated next. Cells were treated with Tm and the protein levels of GCN5 were examined. Increases in BiP proteins were detected 16 hours after Tm treatment, confirming the successful induction of UPR (Figure 5A). A drop in GCN5 protein levels was first observed 8 hours after treatment and remained low up to 24 hours, indicating that the expression of GCN5 was negatively regulated by UPR (Figure 5A). Decreases in the levels of PCAF proteins were also detected at 16 and 24 hours post-treatment (Figure 5A). The involvement of GCN5 for XBP-1S activity during UPR was studied by examining the expression of endogenous XBP-1S target genes, including BiP, CHOP, EDEM, XBP-1, and Erdj4, using qRT-PCR. Cells were transfected with a mock or a GCN5 expression vector, followed by the treatment of Tm. Elevated mRNA levels of XBP-1S target genes were detected after Tm incubation (Figure 5B). Ectopic expression of GCN5 significantly inhibited the expression of all five genes (Figure 5B). An identical set of assays was performed using thapsigargin (Tg) as a UPR inducer and similar results were observed (Figure 5B). To determine the effects of GCN5 on DNA binding of XBP-1S under UPR, chromatin immunoprecipitation (ChIP) assays were performed to examine the abundance of XBP-1S on its target genes. Increased binding between XBP-1S and the promoters of BiP, CHOP, and EDEM genes was detected after Tm treatment (Figure 5C). Overexpression of GCN5 significantly inhibited the enrichment of XBP-1S to its target genes (Figure 5C). Using Tg as an alternative UPR inducer, an identical set of ChIP experiments was carried out and similar inhibitory effects of GCN5 on XBP-1S DNA binding were observed (Figure 5D).

### Acetylation of XBP-1S by GCN5 does not affect the activity of XBP-1S

**Figure 6:**
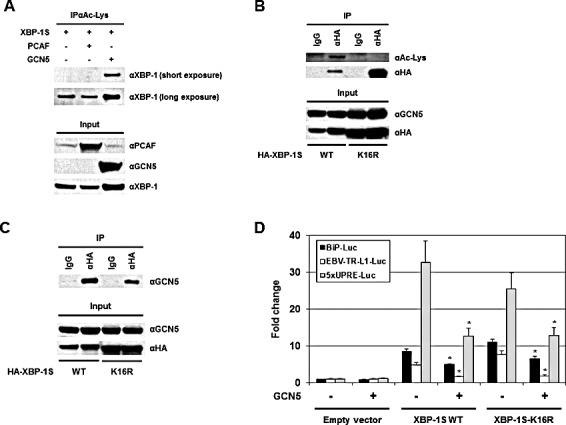
Acetylation of XBP-1S has no effect on XBP-S mediated activation of UPR genes (A) 293T cells were transfected with a XBP-1S and an indicated HAT plasmids (i.e. PCAF or GCN5), followed by IP with an anti-acetylated lysine antibody (i.e. αAc-Lys). The immunoprecipitated complexes and the protein inputs were analyzed by Western blotting. (B) Cells were co-transfected with a GCN5 and a HA-tagged XBP-1S plasmids (i.e. XBP-1S WT and XBP-1S-K16R mutant). IP was performed using an anti-HA antibody. Normal IgG (IgG) was used as a negative control. The immunoprecipitated complexes were analyzed by Western blotting with an anti-acetylated lysine and an anti-HA antibodies. (C) Cell lysates prepared from the transfected cells (GCN5/XBP-1S WT or GCN5/XBP-1S-K16R) were analyzed by IP (αHA antibody), followed by immunoblotting (αGCN5 antibody). (D) Luciferase reporter assays were carried out by transfecting cells with the plasmids as indicated. *P<0.05 vs controls (i.e. cells transfected with the XBP-1S WT and XBP-1S-K16R expression plasmids, respectively).

Protein acetylation is an important post-translational modification that affects a large number of proteins. The significance of histone acetylation in the modification of chromatin structure and gene regulation is well established. A growing number of non-histone proteins, such as transcription factors, have been identified as acetylation targets and their functions can be regulated by acetylation as well [46-48]. Therefore, it may be possible that GCN5 regulates the activity of XBP-1S by acetylation. Ectopically expressed XBP-1S was found to be acetylated at lysine residues by an unknown endogenous HAT(s) (Figure 6A, long exposure). GCN5 was a candidate HAT that could acetylate XBP-1S endogenously since overexpression of GCN5 resulted in acetylation of XBP-1S at lysine residues (Figure 6A). No effects on XBP-1S acetylation was detected when PCAF was overexpressed (Figure 6A). To assess the importance of XBP-1S acetylation in regulating its activity, site-directed mutagenesis was performed to mutate all sixteen lysine residues in XBP-1S to arginine. We first confirmed that this XBP-1S mutant, XBP-1S-K16R, could not be acetylated by GCN5 (Figure 6B). However, XBP-1S-K16R mutant still could bind to GCN5 (Figure 6C). Reporter assays were carried out next and the results showed that XBP-1S-K16R still maintained its transactivating ability to activate BiP, EBV LMP1, and 5×UPRE promoters (Figure 6D). Interestingly, GCN5 was still able to suppress the activity of XBP-1S-K16R, although the XBP-1S mutant could no longer be acetylated by GCN5 (Figure 6D). We conclude that XBP-1S acetylation by GCN5 may not have significant effects on the transactivating activity of XBP-1S.

XBP-1S is a very unstable protein and its half-life is estimated to be around 22 min [49]. We noticed significant increases in protein levels of XBP-1S in GCN5-overexpressing cells (Figures 3D and E). We investigated the impact of GCN5 on the stability of XBP-1S using cycloheximide (CHX), an inhibitor of protein synthesis. In the mock and PCAF-transfected cells, the levels of XBP-1S proteins started to decrease 3 min after CHX treatment and were almost completely diminished at 27-min post-treatment (Figure S2A). In contrast, higher levels of XBP-1S proteins were observed in GCN5-expressing cells and the XBP-1S levels remained unchanged even after 27-min CHX treatment (Figure S2A), demonstrating that overexpression of GCN5 enhanced the stability of XBP-1S. We observed that XBP-1S-K16R mutant was more stable than the wild-type XBP-1S (Figure S2B). In addition, overexpression of GCN5 failed to increase the stability of XBP-1S-K16R mutant proteins (Figure S2B). It has been shown that XBP-1U complexes with XBP-1S and translocates XBP-1S from nucleus into cytoplasm, leading to proteasome-mediated degradation of XBP-1S [6]. We co-transfected cells with HA-tagged XBP-1S and an indicated plasmid (i.e. XBP-1U, PCAF, or GCN5) (Figure S2C). An increase in the protein level of nuclear HA-XBP-1S was detected when GCN5 was overexpressed, while the cytoplasmic amount of HA-XBP-1S remained unchanged (Figure S2C). The GCN5-mediated nuclear retention of XBP-1S was re-confirmed by immunofluorescence (Figure S2D). This result suggests that GCN5 overexpression prevents protein degradation of XBP-1S by retaining it within the nucleus.

Protein ubiquitination mainly occurs on lysine residues of target proteins and results in proteasome-mediated protein degradation [50]. We suspected that the degradation of XBP-1S might be mediated by this ubiquitin-proteasome pathway. The protein levels of wild-type XBP-1S increased after treatment with a proteasome inhibitor, MG132, and overexpression of GCN5 partially inhibited proteasome-mediated degradation (Figure S3A). Increased protein stability was observed in XBP-1S-K16R mutant, which could no longer be ubiquitinated (Figure S3B). Collectively, our data suggested that the stability of XBP-1S protein was regulated by ubiquitination and acetylation at the lysine residues.

### HAT activity of GCN5 is not required for repression of XBP-1S-mediated transcription

**Figure 7:**
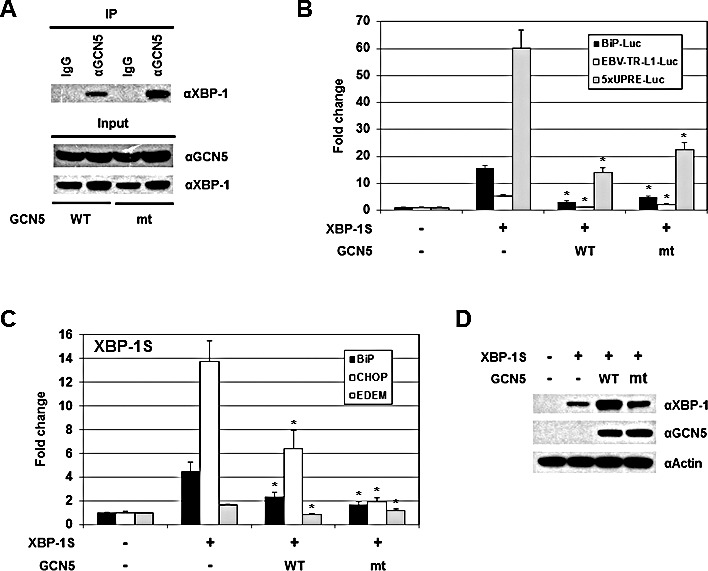
The HAT activity of GCN5 is not required for the inhibition of XBP-1S-mediated transcription (A) IP was performed with an anti-GCN5 antibody and the cell lysates prepared from the transfected cells. IP using the normal IgG (IgG) was used as a control. The immunoprecipitated complexes and the protein inputs were analyzed by Western blotting. (B) Luciferase reporter assays were performed using a specific Luc reporter plasmid (i.e. BiP-Luc or EBV-TR-L1-Luc) and the indicated expression vectors (i.e. XBP-1S, GCN5 WT, and GCN5 mt). (C) MCF7 cells were co-transfected with a XBP-1S expression vector and an indicated GCN5 plasmid (i.e. GCN5 WT or GCN5 mt). ChIP was carried out followed by quantitative PCR to quantify the binding of XBP-1S to the BiP, CHOP, and EDEM promoters. Cells transfected with an empty vector and a XBP-1S plasmid was used as control. *P<0.05 versus controls. (D) The expression levels of XBP-1S in the transfected MCF7 cells were analyzed by immunoblotting. Actin was used as a loading control.

Since acetylation of XBP-1S by GCN5 showed little, if any, effects on its activity, we would like to determine the requirement of GCN5 HAT activity in regulation of XBP-1S. We generated an enzymatically inactive mutant of GCN5, GCN5-Y260A/F261A, as previously described [51]. In a luciferase reporter assay, overexpression of wild-type GCN5 led to a 2.5-fold increase in the activity of HTLV promoter (Figure S4, GCN5 WT). In contrast, GCN5-Y260A/F261A mutant failed to activate the HTLV promoter, confirming the loss of GCN5 HAT activity (Figure S4, GCN5 mt). Loss of the HAT activity in GCN5 did not disrupt its interaction with XBP-1S as demonstrated by IP analysis (Figure 7A, GCN5 mt). In cell-based luciferase assays, overexpression of GCN5 HAT mutant still exhibited strong inhibition on XBP-1S-mediated transcription, including BiP, EBV LMP1, and 5×UPRE (Figure 7B, GCN5 mt). We continued to investigate the impact of GCN5 on the binding between XBP-1S and its target genes. The results obtained from ChIP-quantitative polymerase chain reaction (PCR) experiments showed that overexpression of wild-type GCN5 significantly blocked XBP-1S binding to the promoters of BiP, CHOP, and EDEM genes (Figure 7C, GCN5 WT), and the GCN5 HAT mutant also exhibited a similar pattern of inhibition (Figure 7C, GCN5 mt). It was noted that overexpression of wild-type GCN5 and GCN5 HAT mutant enhanced the protein levels of XBP-1S (Figures S2 and 7D). Even with the increased amounts of XBP-1S proteins in GCN5 WT- and GCN5 HAT mt-expressing cells, XBP-1S-mediated transcription was still significantly inhibited (Figures 7B-D). These results suggest that GCN5 prevents the recruitment of XBP-1S to its target genes and the HAT activity of GCN5 is not required for this inhibitory mechanism.

### GCN5 blocks the recruitment of PCAF to the XBP-1S target genes by disrupting the PCAF-XBP-1S interaction

**Figure 8:**
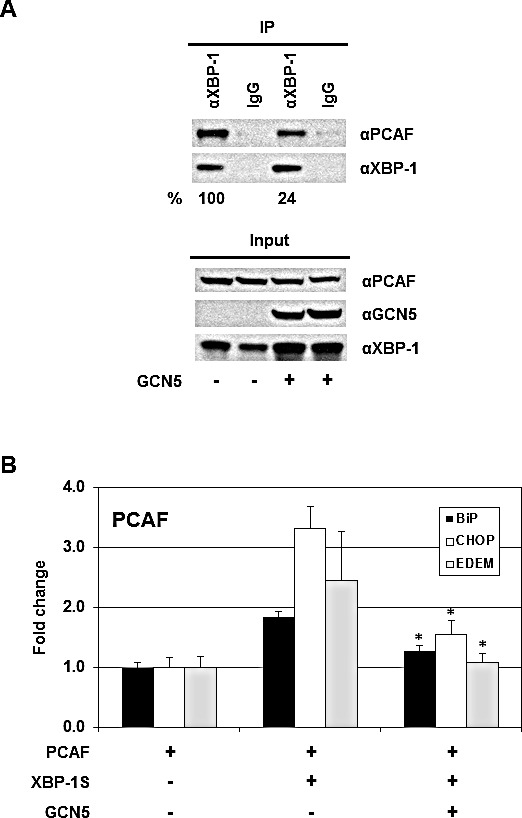
GCN5 competes with PCAF in binding to XBP-1S and inhibits the recruitment of PCAF to the XBP-1S target genes (A) 293T cells were co-transfected PCAF and XBP-1S expression vectors with or without a GCN5 plasmid. IP was performed using an anti-XBP-1S antibody, followed by immunoblotting with anti-PCAF or anti-XBP-1 antibodies. The amounts of PCAF and XBP-1S proteins immunoprecipitated by an anti-XBP-1 antibody were quantified as described under “Materials and Methods.” The XBP-1S protein precipitated in the IP against XBP-1 was used as the input to normalize the amount of PCAF protein detected in the IP. The protein inputs were also analyzed by Western blotting. (B) MCF7 cells were co-transfected with the indicated expression plasmids. ChIP was carried out followed by quantitative PCR to quantify the abundance of PCAF on the BiP, CHOP, and EDEM promoters. Cells only transfected with a PCAF vector were used as a negative control. *P<0.05 versus controls (i.e. cells co-transfected with XBP-1S and PCAF vectors).

Domain mapping demonstrates that both GCN5 and PCAF interact with XBP-1S by binding to the same region of XBP-1S (i.e. the C-terminal transactivation domain; Figures 1D and E) [42]. Thus, it is possible that GCN5 may compete with PCAF in associating with XBP-1S. The impact of GCN5-overexpression on the PCAF-XBP-1S interaction was examined by IP. A 4-fold decrease in the binding between PCAF and XBP-1S was detected in the GCN5 overexpressing cells (Figure 8A). We further investigated the effect of GCN5 on the recruitment of PCAF to the target genes of XBP-1S. In agreement with our previous study, PCAF was recruited to BiP, CHOP, and EDEM genes by XBP-1S (Figure 8B) [42]. However, such XBP-1S-mediated recruitment of PCAF was significantly inhibited when GCN5 was overexpressed (Figure 8B). Taken together, we conclude that GCN5 disrupts PCAF-XBP-1S interaction and inhibits the recruitment of PCAF to XBP-1S target genes.

## DISCUSSION

**Figure 9:**
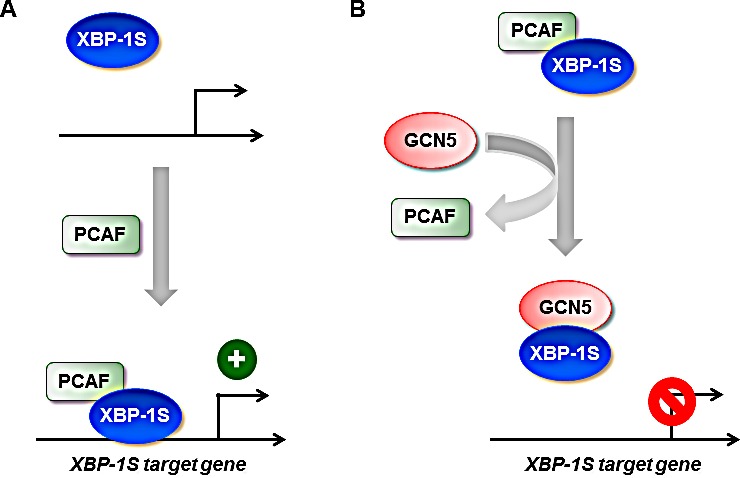
Regulation of XBP-1S activity by HATs (A) XBP-1S interacts and recruits its activator, PCAF, to the target genes of XBP-1S. (B) GCN5 disrupts the formation of XBP-1S-PCAF complexes and prevents the recruitment of XBP-1S to its target genes.

Besides their roles in gene regulation, HATs also have been shown to be involved in tumorigenesis [43, 52-54]. HATs are divided into five families, including GCN5-related N-acetyltransferases (GNATs), MYST (for ‘MOZ, Ybf2/Sas3, Sas2 and Tip60’)-related HATs, p300/CBP HATs, general transcription factor HATs, and nuclear hormone-related HATs [43]. PCAF and GCN5 belong to the same GNAT family and are highly homologous proteins, which share ~73% identity in the amino acid sequences [43]. As expected, both proteins exhibit identical or similar biological functions on their protein substrates [43, 48]. We previously showed that PCAF binds to XBP-1S and stimulates the activity of XBP-1S [42]. In this study, we identify GCN5 as another XBP-1S binding protein and further demonstrate that both PCAF and GCN5 bind to the same transactivation domain of XBP-1S (Figure 1) [42]. This observation raises the possibility that GCN5 may behave like PCAF and function as an activator of XBP-1S. To our surprise, GCN5 was found to potently inhibit the XBP-1S-mediated transcription (Figures 3 and 4). Overexpression of GCN5 almost completely blocks the XBP-1S-dependnt expression of a viral oncoprotein, EBV LMP1, suggesting the potential anti-cancer activity of GCN5 (Figures 3D and E). GCN5 blocks the binding between XBP-1S and the promoters of its target genes (Figures 5C, 5D, and 7C). Furthermore, GCN5 competes with PCAF in binding to XBP-1S and interferes with the XBP-1S-dependent recruitment of PCAF to XBP-1S target genes (Figure 8). Based on our results, we propose a molecular mechanism of XBP-1S, which is positively and negatively regulated by PCAF and GCN5, respectively (Figure 9).

Involvement of GCN5 and GCN5-containing protein complex, Spt-Ada-Gcn5 acetyltransferase (SAGA), in UPR has been reported previously. In yeast, a *Δgcn5* strain failed to induce UPR, indicating a positive and an essential role of GCN5 for UPR [55]. However, it is noted that yeast only has one GCN5 type HAT and does not contain PCAF [43]. In addition, the yeast GCN5 (439 a.a.) is much shorter than human GCN5 (837 a.a.). It only contains the acetyl transferase and bromo domains but missing the N-terminal region (i.e. PCAF homology domain), which is present in vertebrate GCN5 (Fig. 2A) [43]. The addition of PCAF homology domain may have an impact on the activity of human GCN5, which could help to explain the functional difference between yeast and human GCN5 in regulating UPR. In addition, it has been shown that SAGA complex can be recruited, for example, by ATF6α, to the promoters of UPR genes and its role in regulation of UPR gene expression is suggested [56-58]. However, none of the studies has established a direct connection between SAGA and XBP-1S-mediated transcription. Based on the data presented in our study, it is possible that GCN5 or SAGA may be responsible for the activation of XBP-1S-independent UPR genes.

Expression of PCAF and GCN5 was examined under UPR. Treatment with Tm was found to down-regulate the expression of both proteins (Figure 5A). This observation suggests a limited physiological role of PCAF, an activator of XBP-1S, in regulating the activation of XBP-1S target genes during UPR. In contrast, the UPR-induced repression of GCN5, an inhibitor of XBP-1S, may indicate a potential role of GCN5 in UPR. We found that overexpression of GCN5 significantly inhibits the Tm- and Tg-induced gene expression, such as BiP, CHOP, and EDEM, by blocking the recruitment of XBP-1S to the promoters (Figures 5B-D). XBP-1S is mainly synthesized under ER stress. However, under the ER stress-free condition, the presence of XBP-1S is also detected in certain cell lines [10]. Interaction between endogenous GCN5 and XBP-1S was detected under normal condition (Figure 1C). Treatment with an UPR inducer (i.e. Tm) resulted in disruption of the endogenous GCN5-XBP-1S protein interaction, although an increase in the level of XBP-1S proteins was observed (Figure 1C). These results suggest a physiological function of GCN5 in regulating the expression of XBP-1S target genes.

Although both PCAF and GCN5 bind to the same C-terminal region of XBP-1S, these two proteins exhibit different effects on the acetylation and DNA binding of XBP-1S. Domain study on PCAF and GCN5 demonstrated that different regions of PCAF and GCN5 were required for association with XBP-1S (Figure 2). This observation may help explain the functional difference between PCAF and GCN5 on XBP-1S. Overexpression of GCN5 results in acetylation of XBP-1S at its lysine residues (Figure 6A). The acetylation of XBP-1S leads to enhancement in XBP-1S protein stability and changes the subcellular distribution of XBP-1S (Figure S2A, C, and D). XBP-1S with a very short half-life is not a stable protein and is degraded through the proteasome-mediated pathway [6, 49]. It is possible that XBP-1S may be ubiquitinated at its lysine residues by an unknown E3 ubiquitin ligase(s) and the ubiquitinated XBP-1S may later be directed to proteasomes for degradation. GCN5 may acetylate on the same lysine residues which can also be ubiquitinated, and therefore, protect XBP-1S from ubiquitination and degradation. In agreement with this hypothesis, increased protein stability was detected in XBP-1S-K16R, which was also more resistant to proteasome-mediated protein degradation (Figure S2A,2B, and S3). No detectable acetylation of XBP-1S was observed when PCAF was overexpressed (Figure 5A). As expected, PCAF had no effects on either protein stabilization or nuclear-cytoplasmic distribution of XBP-1S (Figure S2A and C).

We performed ChIP assays and demonstrated that GCN5 overexpression consistently inhibited the binding between XBP-1S and its target genes *in vivo* (Figures 5C, 5D, and 7C). On the contrary, PCAF showed little activation or no effects on the DNA binding of XBP-1S (Figure S5). Although PCAF and GCN5 share high identity with each other and bind to the same domain of XBP-1S (Figures 1D and E) [42], these two proteins certainly interact with XBP-1S in a different fashion. This possibility was supported by the domain mapping of PCAF and GCN5. GCN5 was found to associate with XBP-1S through its C-terminus, while both N- and C-terminal regions of PCAF could bind to XBP-1S (Figure 2). Future investigation is required to reveal the structural differences of PCAF-XBP-1S and GCN5-XBP-1S complexes to provide molecular insight into the functional effect caused by PCAF and GCN5.

Protein acetylation is an important post-translational modification affecting a large number of histone and non-histone proteins. The significance of histone acetylation in the modification of chromatin structure and gene transcription regulation has been well recognized. A growing number of non-histone proteins have been identified as acetylation targets. Interestingly, many non-histone proteins (such as p53, nuclear factor-κB, c-Myc, and hypoxia-inducible factor-1α) targeted by acetylation are relevant for tumorigenesis and cancer cell proliferation [59]. Acetylation can stimulate or inhibit the transactivating ability of the transcription factors by affecting their DNA binding activities [60-62]. GCN5 has been reported to acetylate transcription factors Ifh1 and SWI/SNF and reduce their DNA binding ability [63, 64]. ChIP analysis presented in our study demonstrates that GCN5 also inhibits XBP-1 DNA binding *in vivo* (Figures 5C, 5D, and 7C). Surprisingly, the HAT activity of GCN5 is not required for the inactivation of XBP-1S (Figure 7C). This interesting finding suggests that GCN5 may inhibit the transactivation of XBP-1S simply through protein-protein interaction. Subcellular localization of transcription factors can also be regulated by acetylation. Acetylation of hepatocyte nuclear factor-4 (HNF-4) by CREB-binding protein is found to be crucial for nuclear retention of HNF-4 [46]. Here we also observe that GCN5 exhibits a similar effect on XBP-1S and causes the accumulation of XBP-1S in the nucleus (Figure S2). XBP-1S is known to form a heterodimer with XBP-1U and translocate from nucleus into cytoplasm, resulting in proteasome-dependent degradation [6]. Therefore, acetylation by GCN5 retains XBP-1S within the nucleus and stabilizes XBP-1S proteins (Figure S2).

The involvement of UPR/XBP-1S in cancer has been reported by several groups. An anti-cancer approach has been proposed by targeting UPR/XBP-1S [65-67]. Rapid growth of tumor cells is associated with hypoxia and glucose deprivation due to inadequate vascularization in solid tumors. These pathophysiological conditions trigger UPR and induce the activation of XBP-1, which helps cancer cells to survive under such harsh cytotoxic microenvironments [11, 12]. IRE1, which removes 26 nucleotides from the open reading frame of XBP-1 mRNA, is required for the production of XBP-1S upon activation of UPR [4]. Blockade of XBP-1S synthesis using IRE1-inhibiting compounds, including STF-083010, toyocamycin, and MKC-3946, has been demonstrated to be a promising therapeutic option against MM [13-15]. Triple-negative breast cancer (TNBC) is a form of breast cancer that does not express oestrogen receptor, progesterone receptor, and HER2 [68]. TNBC is highly aggressive with limited treatment options since most chemotherapies target one of the three receptors [68]. A recent study indicates a high-level expression of XBP-1S in TNBC and knockdown of XBP-1 effectively blocks TNBC cell growth and invasiveness [69]. In this study, we identify GCN5 as an inhibitor of XBP-1S. GCN5 exhibits potential anti-cancer activity by blocking the expression of an oncogene, EBV LMP1, which is regulated by XBP-1S. Future investigation will be carried out to determine the anti-cancer activity of GCN5 on MM and TNBC.

Infection by various viruses can also trigger UPR in their host cells. Some viruses, such as JEV and DV, use ER of host cells as the primary site of glycoprotein synthesis, genomic RNA replication, and virus particle maturation, and thus trigger ER stress as well as UPR [18, 24]. In the other cases, some viral proteins, such as HCMV US11 and HTLV-1 Tax, traffic to ER and Golgi complex of host cells and induce UPR [22, 38]. In many cases, viruses modulate UPR to attenuate anti-viral defenses of host cells and facilitate viral gene expression and replication. We have demonstrated the requirement of XBP-1S in the gene regulation of HTLV-1 and EBV LMP1 [34, 35]. Besides our studies, other groups have shown the involvement of XBP-1S in reactivation of KSHV [70] and replication of IV [33], suggesting that XBP-1S may serve as a potential target for development of therapeutics against these viruses. Many anti-viral drugs target viral proteins. However, due to high mutation rates of viruses, treatment with drugs of this category results in the selection of resistant viral strains. Since XBP-1S is a cellular factor, it is less likely that resistant strains will arise using anti-viral therapeutics against XBP-1S.

## METHODS

### Cells, siRNAs, plasmids, and chemicals

HEK293, 293T, and MCF7 cells were obtained from American Type Culture Collection. EBV-infected NPC-TW01 cells were described in our previous study [35]. The GCN5 siRNAs (siGCN5-3: 5′-CTCCATTTGAGAAACCTAATA-3′ and siGCN5-4: 5′-CCGCGGCATCATCGAGTTCCA-3′) and the siRNAs against firefly GL2 and GL3 luciferases were purchased from Qiagen. Human XBP-1S and XBP-1U expression plasmids and the firefly luciferase reporter plasmid, EBV-TR-L1-Luc, were described previously [34, 35]. The expression plasmids of human PCAF (accession #: BC060823) and GCN5 (accession #: BC105977) were obtained from Open Biosystems. To generate the green fluorescent protein (GFP) tagged PCAF and GCN5 truncations, the PCAF and GCN5 cDNAs were used as the templates for PCR amplification. The amplified DNA fragments were subcloned into a pAcGFP-C1 vector (Clontech). The XBP-1S-K16R mutant plasmid was generated by site-directed mutagenesis (Stratagene) to replace all sixteen lysine residues in XBP-1S with arginine. The HAT-inactive GCN5 mutant, GCN5-Y260A/F261A, was generated as previously described (28). The plasmids containing a series of HA-tagged XBP-1 deletions were generous gifts from Dr. Hiderou Yoshida [6]. The firefly luciferase reporter plasmids, HTLV-Luc, BiP-Luc, and 5×unfolded protein response element-Luc (5×UPRE-Luc) (i.e. p5×UPRE-GL3) were kindly provided by Dr. Arnold Rabson and Dr. Kazutoshi Mori, respectively [45, 71, 72]. Tm (Sigma), Tg (Sigma), MG132 (Calbiochem), and BFA (Sigma) were dissolved in DMSO. To induce UPR, cells were treated with 0.1 μg/ml BFA for 24 hours, 10 μg/ml Tm for 16 hours, or 300 nM Tg for 4 hours.

### Transient transfection and luciferase assays

Transient transfections of DNA plasmids into HEK293, 293T, and MCF7 cells were performed using FuGENE 6 (Roche) according to the manufacturers' instructions. To perform the cell-based overexpression assays, cells were grown to 50-80% confluence in 96-well plates and co-transfected with a luciferase reporter and an expression plasmid. Lipofectamine 2000 Reagent (Invitrogen) was utilized to co-transfect cells with DNA plasmids and siRNAs for the cell-based knockdown experiments. Firefly luciferase activities were measured 48 hours post-transfection using the Bright-Glo assay system (Promega) and the activities were determined using an Infinite 200 multiplate reader (Tecan).

### Co-IP and Western blotting

293T cells were transiently co-transfected with indicated expression plasmids and the cell lysates were prepared two days post-transfection for Co-IP. To get the high levels of ectopic expression, 293T, a highly transfectable derivative of HEK293, was chosen for the Co-IP study. The IP kit was purchased from Roche and Co-IP was performed according to the manufacturers' instructions. The immunoprecipitated complexes were analyzed by Western blotting. Western blotting was carried out according to the standard protocols. The horseradish peroxidase-conjugated secondary antibodies were purchased from Pierce. After antibody incubation, the blots were washed and incubated with SuperSignal West Pico Substrate (Pierce), and the chemiluminescent signal was detected using an x-ray film (Roche). The film was then scanned, and the protein bands were quantified by the GS-800 densitometer (Bio-Rad). The antibodies used in this study include: anti-EBV LMP1 (ETU001, KeraFAST), anti-XBP-1 (sc-7160), anti-BiP (sc-1501), anti-PCAF (sc-8999), anti-lamin B (sc-373918, Santa Cruz Biotechnology), anti-acetylated lysine (ab21623), anti-GCN5 (ab71965, Abcam), anti-HA (H3663, Sigma), anti-actin (MAB1501, Millipore), and anti-glyceraldehyde 3-phosphate dehydrogenase (GAPDH) (#2118S, Cell Signaling).

### qRT-PCR

Total RNAs of the expression vector-transfected or the Tm/Tg-treated cells were isolated using RNeasy mini kit (Qiagen). One microgram of the total RNAs was converted into complementary DNA (cDNA) using ImProm^TM^-II Reverse Transcription System (Promega). Specific cDNAs were amplified using SYBR Green PCR Master Mix (Applied Biosystems). The primer pairs used in this study include: BiP (5′-GGTGAAAGACCCCTGACAAA-3′ and 5′-GTCAGGCGATTCTGGTCATT-3′), CHOP (5′-CTTCTCTGGCTTGGCTGACT-3′ and 5′-CCCTTGGTCTTCCTCCTCTT-3′), EDEM (5′-AGGTGCTGATAGGAGATGTGG-3′ and 5′-GGATTCTTGGTTGCCTGGTA-3′), ERdj4 (5′-GTCGGAGGGTGCAGGATATT-3′ and 5′-GGTGGTACTTCATGGCCAAC-3′), XBP-1 (5′-GGAGTTAAGACAGCGCTTGG-3′ and 5′-ACTGGGTCCAAGTTGTCCAG-3′), and GAPDH (5′-AACAGCCTCAAGATCATCAGC-3′ and 5′-GGATGATGTTCTGGAGAGCC-3′). GAPDH was used as a control to normalize the cDNA inputs. Amplification and detection of cDNAs were performed using ABI Prism 7500 Thermal-Cycler (Applied Biosystems).

### Quantitative ChIP

ChIP assays were carried out using EZ ChIP kit (Millipore) according to the manufacturer's protocol with some modifications. 293T cells were treated with Tm or Tg prior to cross-linking. DNA fragments at around 200-1000 bp were achieved by sonication with Microson Ultrasonic Cell Disruptor (Misonix). For IP, the indicated antibodies (i.e. anti-XBP-1 or anti-PCAF antibodies) were added to the sheared chromatin individually and incubated at 4°C overnight. The DNA/protein/antibody complex was then pulled down by protein G agarose and the DNA in the complex was purified using QIAquick PCR purification kit (Qiagen). Quantitative-PCR was performed to determine the relative amount of DNA that was immunoprecipitated by anti-XBP-1 or anti-PCAF antibodies in the presence of Tm or Tg. The primer pairs used to amplify the promoter regions of BiP, CHOP and EDEM genes include: BiP (5′-GATGGGGCGGATGTTATCTA-3′ and 5′-CTCTCACACTCGCGAAACAC-3′), CHOP (5′-GACACTACGTCGACCCCCTA-3′ and 5′-GGTTCCAGCTCTGATTTTGG-3′), and EDEM (Epitect ChIP qPCR primers, Qiagen). Cells treated with DMSO served as a negative control. For overexpression, MCF7 cells were co-transfected with the indicated expression vectors two days prior to cross-linking, followed by ChIP-quantitative-PCR as described earlier.

### Statistical analysis

The data shown (including luciferase assays, qRT-PCR, and quantitative ChIP) were analyzed using Student's t test at 5% significance level (*P*<0.05).

## SUPPLEMENTARY MATERIAL AND FIGURES


