# Supplementary material for: GCN5 inhibits XBP-1S-mediated transcription by antagonizing PCAF action
Source: Oncotarget. 2014 Nov 16;6(1):271–87. doi: 10.18632/oncotarget.2773 (PMC4381594; doi:10.18632/oncotarget.2773)
Supplement: Supplementary file 1 [file oncotarget-06-271-s001.pdf]

GCN5 inhibits XBP-1S-mediated transcription by antagonizing PCAF action

Supplementary Material

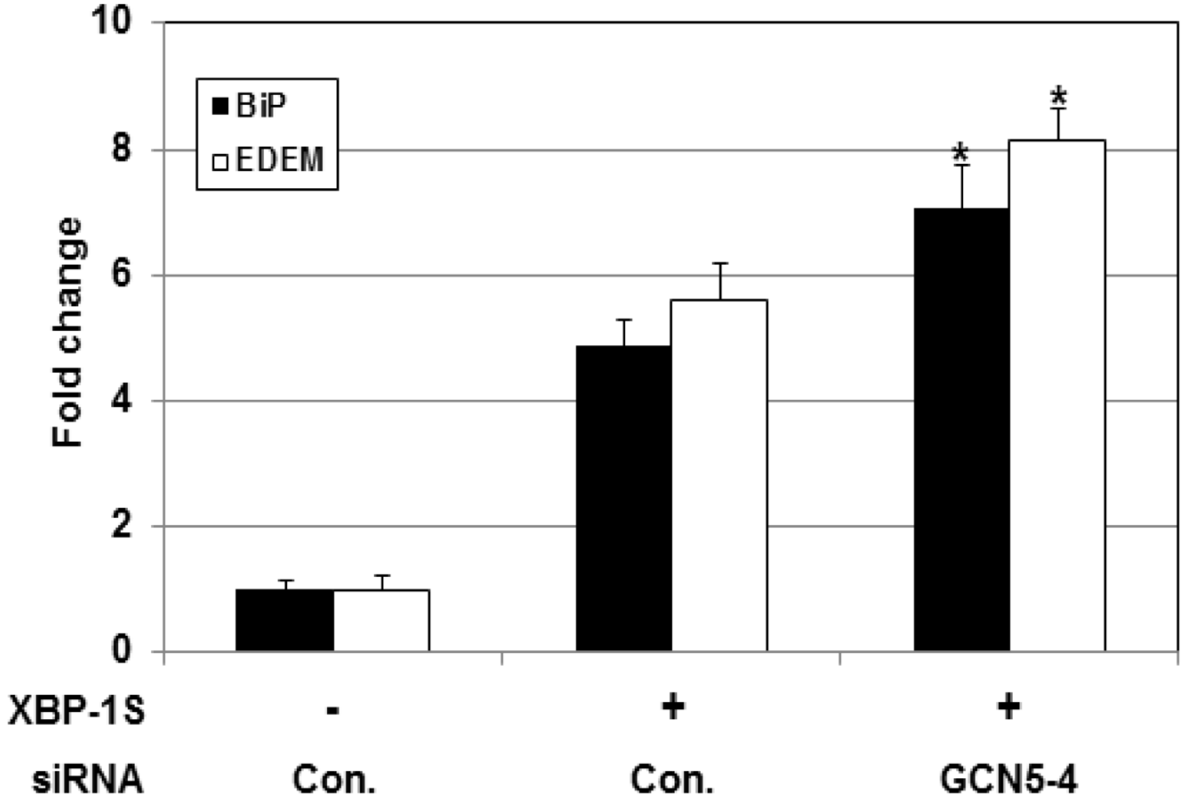

Figure S1: Effects of GCN5 knockdown on the expression of endogenous XBP-1S target genes. 293T cells were co-transfected with a XBP-1S expression vector and an indicated siRNA (i.e. control or GCN5-4 siRNAs). Expression of endogenous BiP and EDEM genes was determined by qRT-PCR. *$P<0.05$ vs ~~negative~~ controls (i.e. cells transfected with a XBP-1S vector and a control non-specific siRNA).

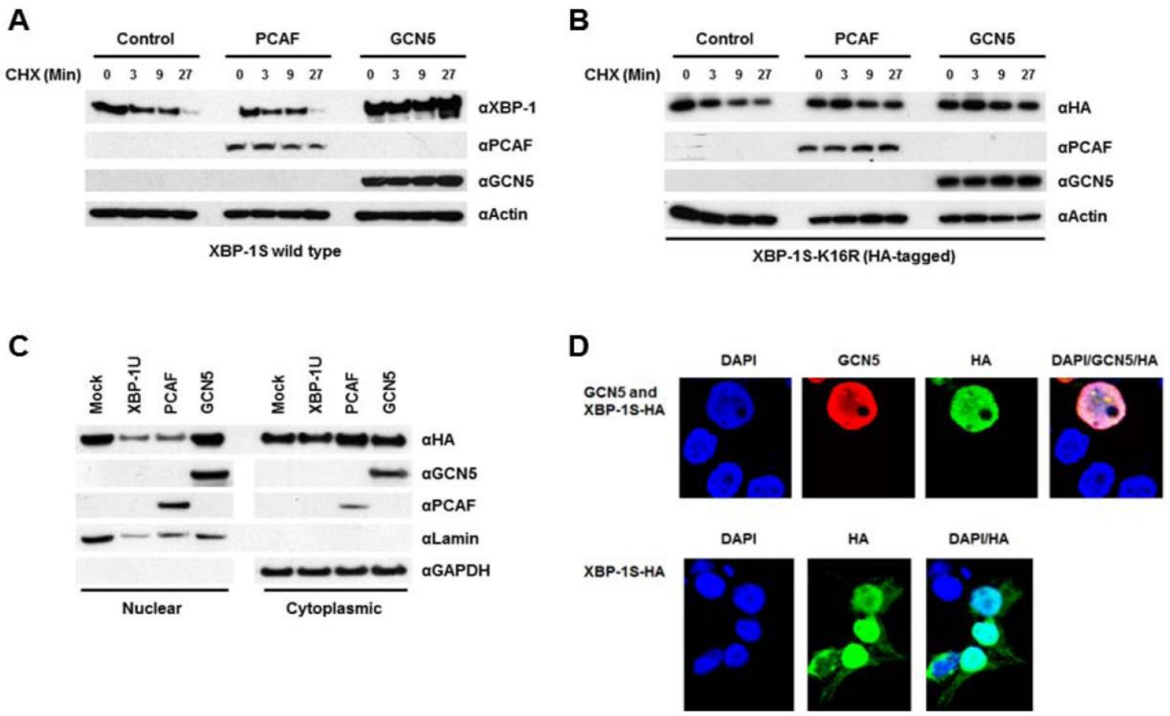

Figure S2: GCN5 regulates the protein stability and subcellular distribution of XBP-1S. 293T cells were transfected with a wild type XBP-1S (A) or a mutant XBP-1S-K16R vector (B), along with an indicated plasmid [i.e. empty (control), PCAF, or GCN5 vectors]. Two days after transfection, the cells were incubated with 100 µg/ml cycloheximide (CHX) for 0, 3, 9, and 27 min, followed by Western blotting. (C) 293T cells were transfected with a HA-XBP-1S vector and an indicated plasmid (i.e. mock, XBP-1U, PCAF, or GCN5 vectors). The nuclear and cytoplasmic fractions of the transfected cells were analyzed by Western blotting. Lamin and GAPDH were utilized as the marker proteins for nuclear and cytoplasmic fractions, respectively. (D) 293T cells were transfected with a HA-XBP-1S and GCN5 expression vectors. Cells transfected with a HA-XBP-1S plasmid were used as a control. Transfected cells were immunostained with anti-HA (green) and anti-GCN5 (red) antibodies. Nuclei were visualized by DAPI.

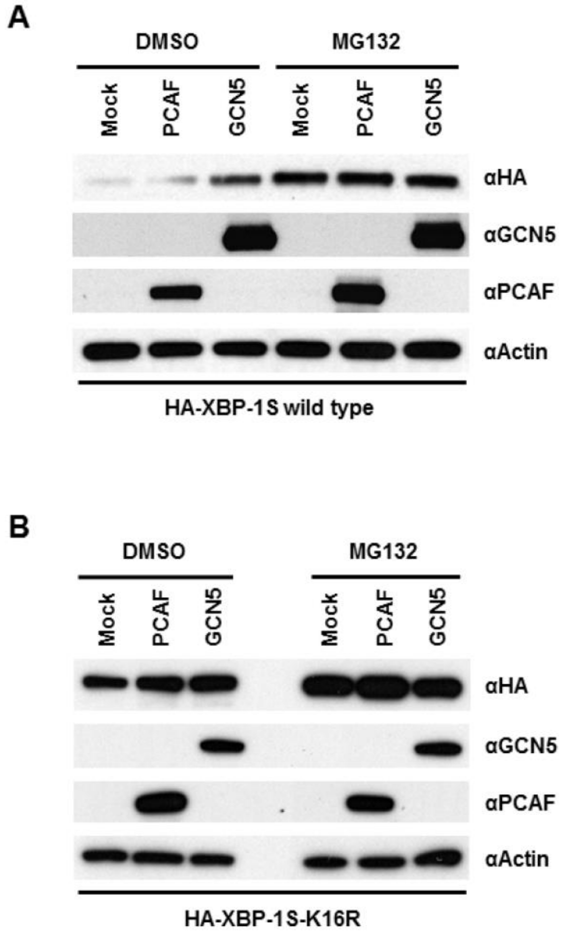

Figure S3: GCN5 regulates the proteasome-mediated degradation of XBP-1S. 293T cells were transiently transfected with a HA-XBP-1S wild type (A) or HA-XBP-1S-K16R (B), along with the indicated expression plasmids. The transfected cells were harvested 2 days after transfection. 16 hours before harvest, cells were treated with 10 µM proteasome inhibitor MG132. Cell lysates prepared from the transfected cells were analyzed by Western blotting.

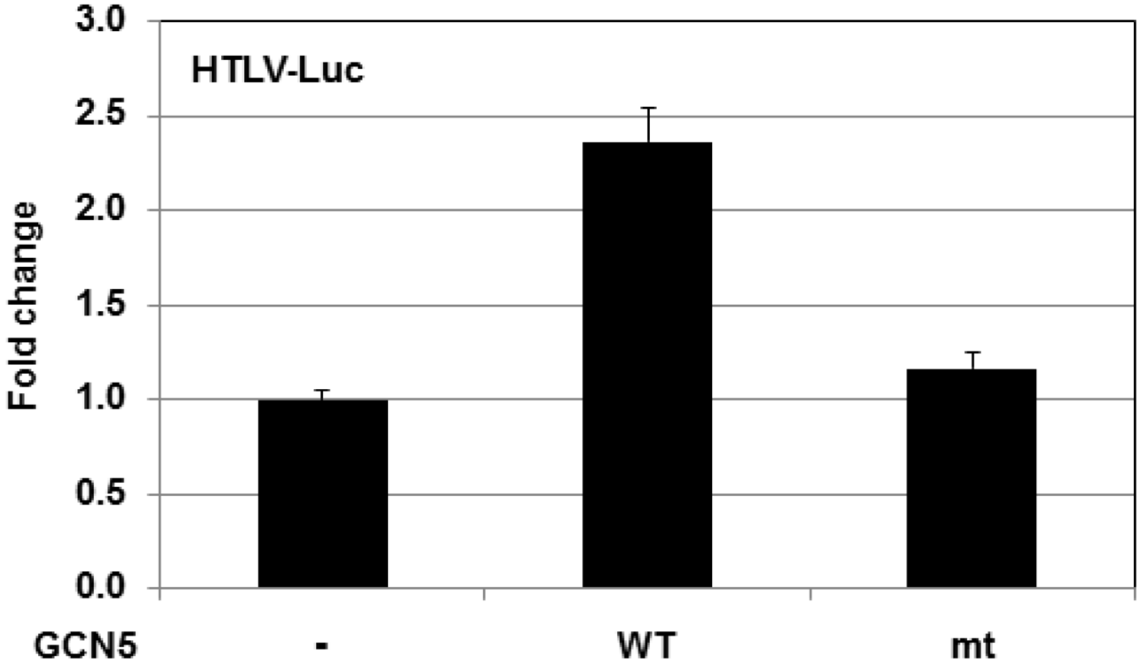

Figure S4: GCN5 HAT mutant does not activate the promoter of HTLV-1. HEK293 cells were transfected with the HLTV-Luc reporter plasmids and an indicated vector [i.e. mock, GCN5 WT, or GCN5 HAT mutant (GCN5 mt) plasmids]. Luciferase activity was measured two days post-transfection.

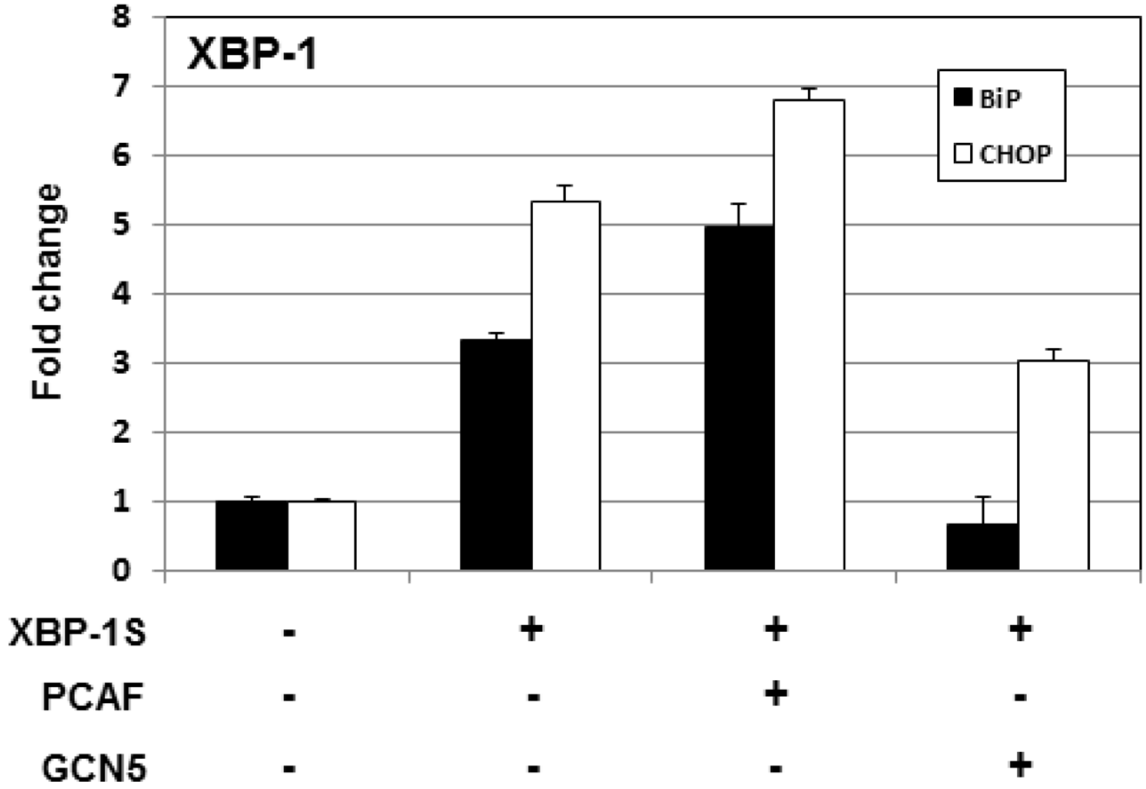

Figure S5: Effects of PCAF on the DNA binding of XBP-1S *in vivo*. MCF7 cells were co-transfected with the indicated expression plasmids. ChIP was carried out followed by quantitative PCR to quantify the abundance of XBP-1S on the BiP and CHOP promoters.
